# Emergent community architecture despite distinct diversity in the global whale shark (*Rhincodon typus*) epidermal microbiome

**DOI:** 10.1038/s41598-023-39184-5

**Published:** 2023-08-07

**Authors:** Michael P. Doane, Michael B. Reed, Jody McKerral, Laís Farias Oliveira Lima, Megan Morris, Asha Z. Goodman, Shaili Johri, Bhavya Papudeshi, Taylor Dillon, Abigail C. Turnlund, Meredith Peterson, Maria Mora, Rafael de la Parra Venegas, Richard Pillans, Christoph A. Rohner, Simon J. Pierce, Christine G. Legaspi, Gonzalo Araujo, Deni Ramirez-Macias, Robert A. Edwards, Elizabeth A. Dinsdale

**Affiliations:** 1 Flinders University, Bedford Park, SA Australia; 2 North Carolina Agricultural and Technical State University, Greensboro, NC USA; 3 San Diego State University, San Diego, CA USA; 4 Lawrence Livermore National Laboratory, Livermore, CA USA; 5 Hopkins Marine Station, Department of Biology, Stanford University, Pacific Grove, CA USA; 6 Australian Centre for Ecogenomics, University of Queensland, St Lucia, QLD Australia; 7 Ch’ooj Ajauil AC, Cancún, Centro Mexico; 8 CSIRO, Brisbane, Australia; 9 Marine Megafauna Foundation, West Palm Beach, FL USA; 10 Department of Biological and Environmental Sciences, Qatar University, Doha, Qatar; 11 Marine Research and Conservation Foundation, Lydeard St Lawrence, Somerset UK; 12 Tiburon Ballena Mexico de Conciencia Mexico, La Paz, Baja California Sur Mexico; 13 Independent Researcher, Quezon City, Philippines

**Keywords:** Ecology, Biodiversity, Community ecology, Ecological networks, Microbial ecology

## Abstract

Microbiomes confer beneficial physiological traits to their host, but microbial diversity is inherently variable, challenging the relationship between microbes and their contribution to host health. Here, we compare the diversity and architectural complexity of the epidermal microbiome from 74 individual whale sharks (*Rhincodon typus*) across five aggregations globally to determine if network properties may be more indicative of the microbiome-host relationship. On the premise that microbes are expected to exhibit biogeographic patterns globally and that distantly related microbial groups can perform similar functions, we hypothesized that microbiome co-occurrence patterns would occur independently of diversity trends and that keystone microbes would vary across locations. We found that whale shark aggregation was the most important factor in discriminating taxonomic diversity patterns. Further, microbiome network architecture was similar across all aggregations, with degree distributions matching Erdos–Renyi-type networks. The microbiome-derived networks, however, display modularity indicating a definitive microbiome structure on the epidermis of whale sharks. In addition, whale sharks hosted 35 high-quality metagenome assembled genomes (MAGs) of which 25 were present from all sample locations, termed the abundant ‘core’. Two main MAG groups formed, defined here as Ecogroup 1 and 2, based on the number of genes present in metabolic pathways, suggesting there are at least two important metabolic niches within the whale shark microbiome. Therefore, while variability in microbiome diversity is high, network structure and core taxa are inherent characteristics of the epidermal microbiome in whale sharks. We suggest the host-microbiome and microbe-microbe interactions that drive the self-assembly of the microbiome help support a functionally redundant abundant core and that network characteristics should be considered when linking microbiomes with host health.

## Introduction

For eukaryotic organisms, the diverse species that makeup their microbiomes are more than just passengers^1^: they affect metabolic and immune processes^2^ and confer physiological functions beyond the host’s innate capabilities, promoting health^3, 4^. These services result from a multitude of interactions between microbes and host cells that become established over time^5^. Yet, microbiome species composition and diversity are highly variable in space and time and across individuals from similar species^6^, often with no apparent consequence to the host. These observations suggest that the function or the services rendered by the microbiome are not attributed simply to the presence or abundance of individual species^7^. The self-organization, or interaction network of microbiome communities, is one explanation for how consistent services that support healthy microbiomes are maintained^8^, however, whether host microbiomes exhibit emergent structure remains an outstanding question.

Self-organization of the microbiome refers to the collective behaviour of the microbial members, captured as population patterns of microbial groups relative to each other, described as co-occurrence: a pattern described as microbiome architecture. Architectural properties (i.e., number of nodes and associated edges or number of clusters formed by interacting microbes) of the microbiome arise in response to ecological and evolutionary processes^9^, which influence the host-microbe and microbe-microbe interactions^7^. Together, these eco-evolutionary processes drive population dynamics of these microbes which result in the microbiome architectural properties. Therefore, the rearrangement of microbial populations can subsequently alter emergent ecosystem functions^10^. As networks are inherently hierarchical^11^, quantifying the different attributes of these levels, such as network complexity, modularity, and individual microbial group interactions, can reveal important ecological insight driving community structure^12^. For instance, fluctuation in microbiome architectural complexity in response to different abiotic stress results in changes to emergent community properties^8^, while microbial organisms that form sub-networks suggest similar environmental preferences^13, 14^. Microbes with many connections to other microbial groups are predicted to be important in niche formation and considered keystone organisms^15^. For these reasons, determining the microbiome's network architecture is fundamental to understanding microbiome structure and function.

In this study, we examine epidermal microbiomes in whale sharks (*Rhincodon typus*) to test our hypothesis that the host-microbiome relationship relies on self-organization of microbiome members for required functions, rather than being dependent on specific taxonomic groups. Whale sharks are an ideal non-model host system to address this fundamental microbiome assembly hypothesis for the following reasons: (1) They are part of the ancient extant vertebrate lineage of Chondrichthyan fishes, which places them at a pivotal evolutionary point for understanding the vertebrate host-associated microbiome relationship; (2) they are one of a few animals species which have a global distribution, occupying tropical/subtropical waters between latitudes 30° N and 35° S^16^; and (3) these animals form predictable seasonal aggregations at specific sites taking advantage of plankton blooms. Using random shotgun metagenomics, we quantified the microbiome architecture from the epidermis of 74 individual whale sharks distributed across five aggregations from around the world, spanning every major ocean basin. Our results indicate that microbiome diversity and composition correspond with aggregation; however, microbiome architecture is a fundamental feature the epidermal microbiome across the globe. In addition, abundant core microbiome members, identified as Metagenome Assembled Genomes (MAGs) discriminated into two main groups, termed Ecogroup 1 and 2 based on potential gene functions, revealing at least two distinct ecological niches within the epidermal microbiome. We predict these distinct functions support important metabolic processes that may help to stablise the microbiome architecture of whale shark.

## Results

**Figure 1:**
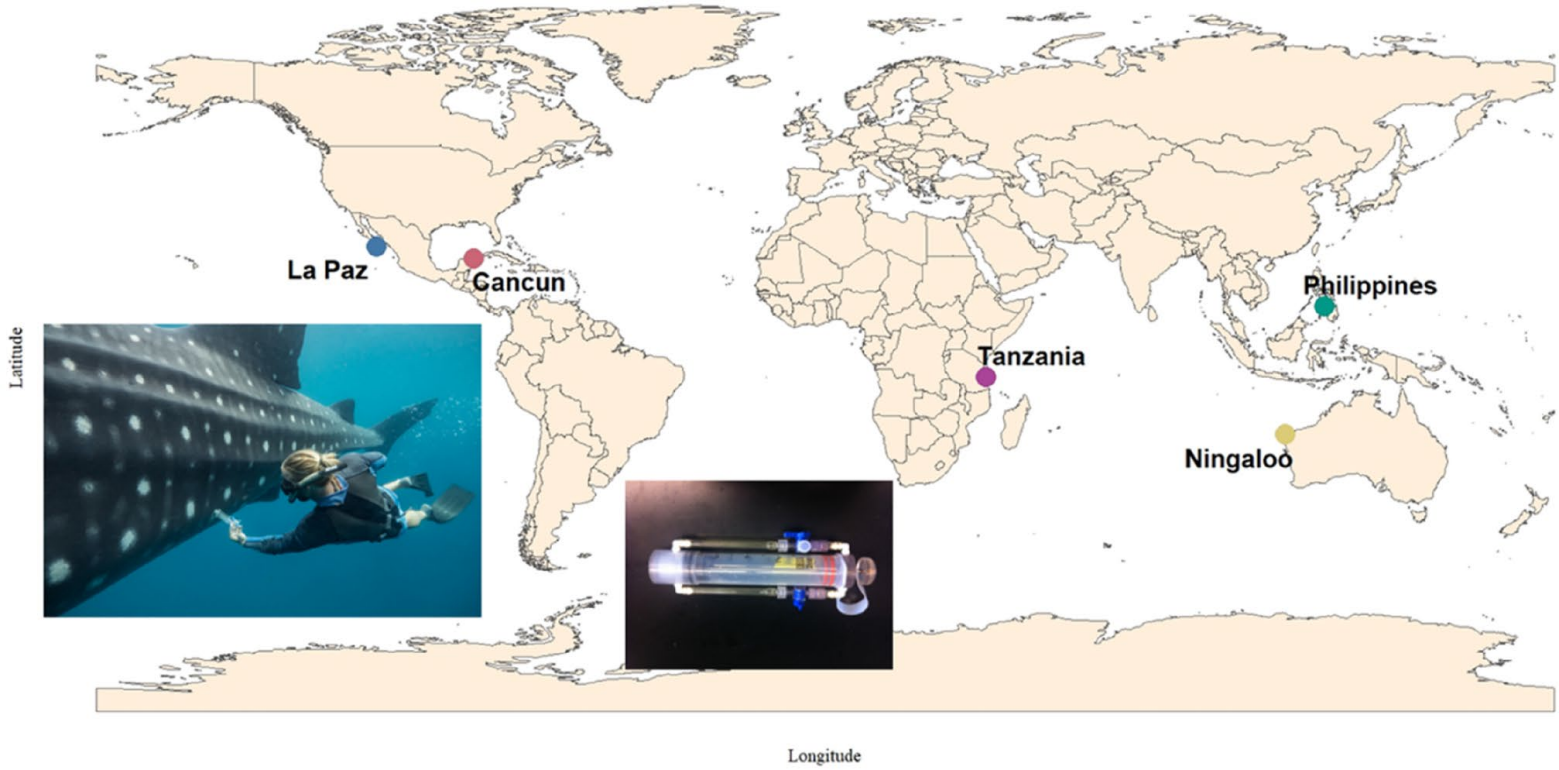
The location and the sampling methods used to analyse the skin microbiome of whale sharks. The sample locations are from aggregation globally distributed and denoted by colored dots. Inserts demonstratre how sampling was performed and the two-way syringe device for flushing sterile seawater over the skin surface of the whale sharks, isolating the epidermal microbiome from the surrounding water column.

Epidermal microbiomes were collected from the dorso-lateral skin surface, in line with the first dorsal fin of 74 whale sharks at five aggregations distributed globally (Fig. 1; SI Table 1). The mean library size ranged from 335,820 (± 46,523) reads at Cancun to 701,829 (± 70,068) at Ningaloo (SI Fig. 1a; SI Table 1). The proportion of reads with taxonomic assignments was lowest at Ningaloo (taxa: 16.5% ± 1.5%) and highest in the Philippines (54.4 ± 1.5%) (SI Fig. 1b).

### Taxonomic diversity structure of whale shark epidermal microbiomes

**Figure 2:**
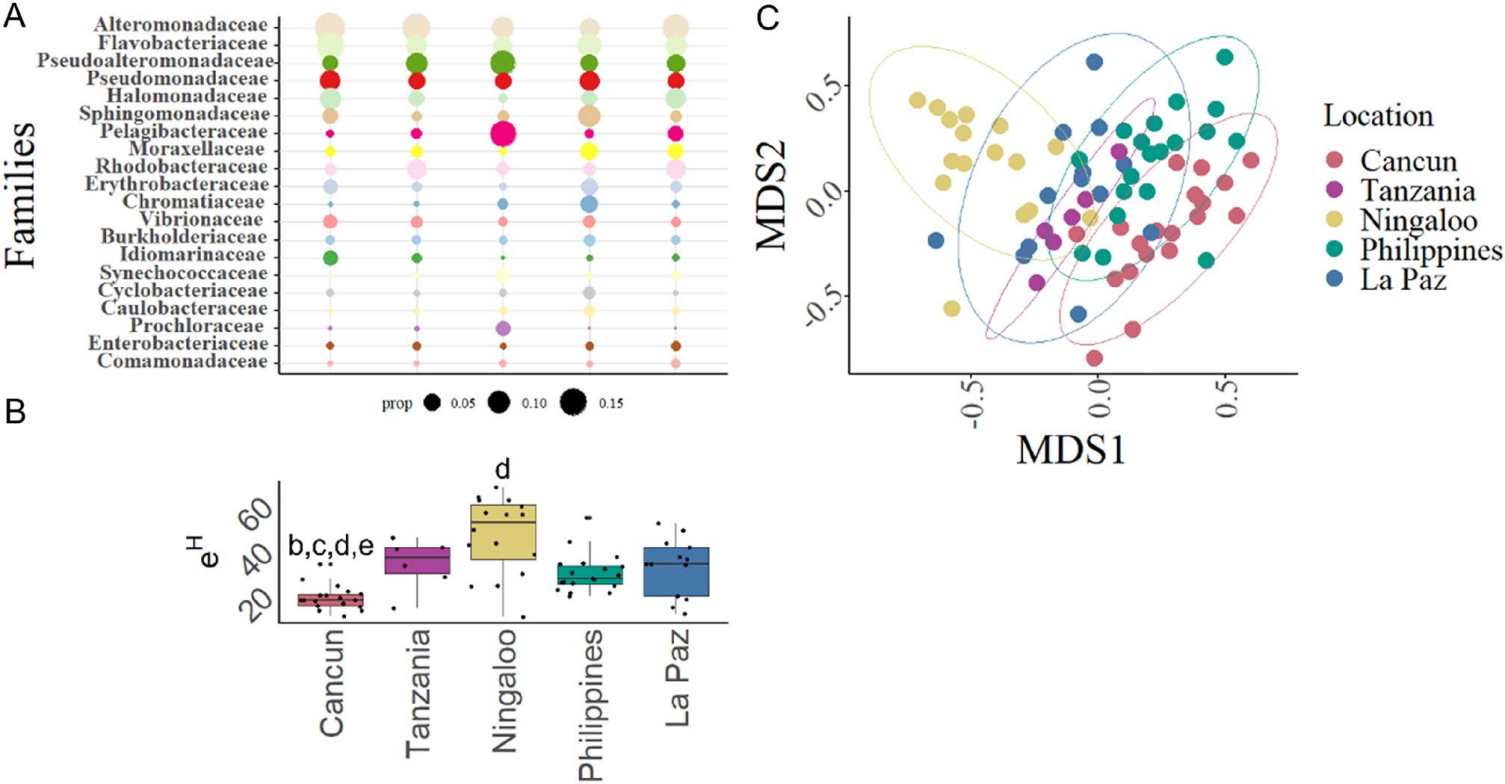
Microbiome taxonomic diversity and compositional patterns across whale sharks at five locations. (**A**) Mean distribution of the 20 most abundance taxa at the family level found across all locations. The size of the dot corresponds to the relative proportion each family. Dot size proportion is indicated below the figure. (**B**) Taxonomic alpha diversity measured as effective number of microbial families. Dots over boxes demonstrate the distribution of diversity values for each location. Letters correspond to the location which comparison is significantly different from at *p* < 0.05. Figure A and B share location labels. (**C**) Whale shark taxonomic microbiome compositional patterns based on Bray–Curtis dissimilarity. Each point represents a metagenomic sample, and the ellipse notes the 95% confidence for sample spread at each location in multivariate space.

The 20 most relative abundant microbial families accounted for 66% to 82% of total proportional abundance (Fig. 2a). Proportionally abundant families included Alteromonadaceae (max: Cancun mean of 18.7 ± 2.9%—min: Philippines 7.8 ± 1.7%), Flavobacteriaceae (max: Cancun 16.1 ± 2.2%—min: Ningaloo 5.0 ± 0.9%); Pseudoalteromonadaceae (max: Ningaloo 11.5 ± 2.8%—min: Cancun 4.5 ± 1.2%) and Pseudomonadaceae (max: Cancun 9.8 ± 1.9%—min: Ningaloo 4.5 ± 0.8%). A few families showed high relative abundance on sharks at one aggregation but low abundance elsewhere; for example, Sphingomonadaceae had a mean of 10.5 (± 1.8) % in the Philippines, and Pelagibacteraceae a mean of 14.6 (± 1.9) % at Ningaloo (Fig. 2a).

Family level effective diversity was significantly different across aggregations (Kruskal–Wallis χ^2^_df = 4_ = 30.02; *p* < 0.01; Fig. 2b; SI Table 2) with Cancun having the least diverse microbiomes (Effective diversity; e^H`^: 20.4 ± 1.21) and Ningaloo the most diverse (47.9 ± 4.25). Richness of microbial families was also significantly different across aggregations (Kruskal–Wallis χ^2^_df = 4_ = 17.1; *p* < 0.01; min: La Paz 338 ± 17.6 families – max: Tanzania 384 ± 4.6 families, SI Fig. 2a; SI Table 3) as was evenness (Kruskal–Wallis χ^2^_df = 4_ = 30.3; *p* < 0.01; Pielou’s evenness; min: 0.51 ± 0.01 at Cancun—max: 0.6 ± 0.02 at Ningaloo SI Fig. 2b; SI Table 4). Therefore, both the number of microbial families and the relative abundance of each microbial family affected the diversity patterns of microbiomes across aggregations.

Compositional patterns of whale shark epidermal microbiomes also varied. We first tested the whale shark microbiomes to water samples collected from each location. Whale shark epidermal microbiomes did vary from the water column (PERMANOVA: Pseudo-F _df = 1, 79_ = 2.43, *p* < 0.05; R^2^ = 0.03, SI Fig. 3). We then analyzed several factors to determine the best predictor of microbiome compositional difference across sharks. Aggregation was the strongest predictor of microbiome variation (PERMANOVA: Pseudo-F _df = 4,69_ = 4.09, *p* < 0.001; R^2^ = 0.19; Fig. 2c). Ocean basin from which the whale shark aggregation occurred was also significant, but with less explanatory power (PERMANOVA: Pseudo-F _2,71_ = 4.52, *p* < 0.001; R^2^ = 0.11). We additionally tested population structure of whale sharks by grouping locations together on well established whale shark population estimations^16^ and found significant groupings, but with much less explained variation (PERMANOVA: Pseudo-F _1,72_ = 6.55, *p* < 0.001; R^2^ = 0.08), relative to the aggregation factor. A pairwise PERMANOVA was performed on aggregation and indicated the taxonomic composition of microbiomes from Cancun whale sharks were different to all other aggregations (*p* < 0.01). La Paz whale shark microbiomes were different to the Philippines and Tanzania (*p* = 0.04), but interestingly, there was no difference between Tanzania, Ningaloo, and Philippine whale shark microbiomes, which are in the Indian ocean region.

**Figure 3:**
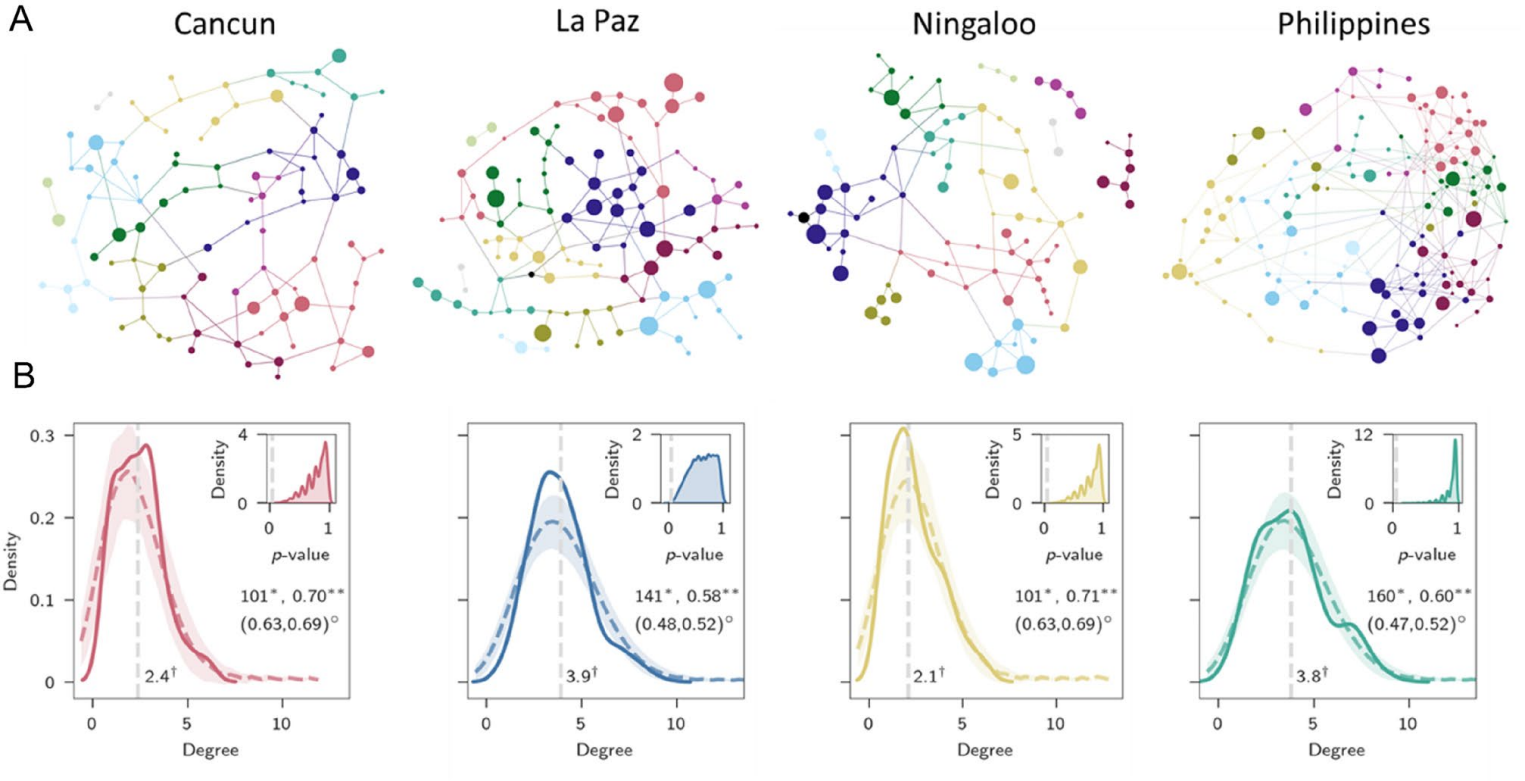
Network structure of microbial taxonomy from whale sharks across the four locations, including Cancun, La Paz, Ningaloo, and Philippines microbiomes. (**A**) Networks were calculated with the SpiecEasi algorithm. Network nodes have been oriented such that nodes with large degrees (more edges) are centralized while nodes with lower degree are peripheral. Color of nodes represents the assigned cluster. (**B**) Degree distribution for microbiome-derived networks compared against modelled degree distribution patterns. Degree distributions for the microbiome-derived networks are signified as a bold line in bi-plots, while the shaded region representes the estimated densities of 5000 generated Erdos–Renyi networks. The dotted vertical line (†) demonstrates the mean degree (number of edges) of all nodes in the microbiome-networks. Inserts within each bi-plot indicate *p*-value distributions of 5000 tests. The dotted line within the *p*-value distribution represents 0.05 cut-off. Symbols in figure include: † mean degree value of whale shark derived network; * the number of nodes in whale shark derived network; ** modularity for whale shark derived networks; °lower and upper modularity values for modelled networks.

### Taxonomic network structure of whale shark epidermal microbiome communities

**Figure 4:**
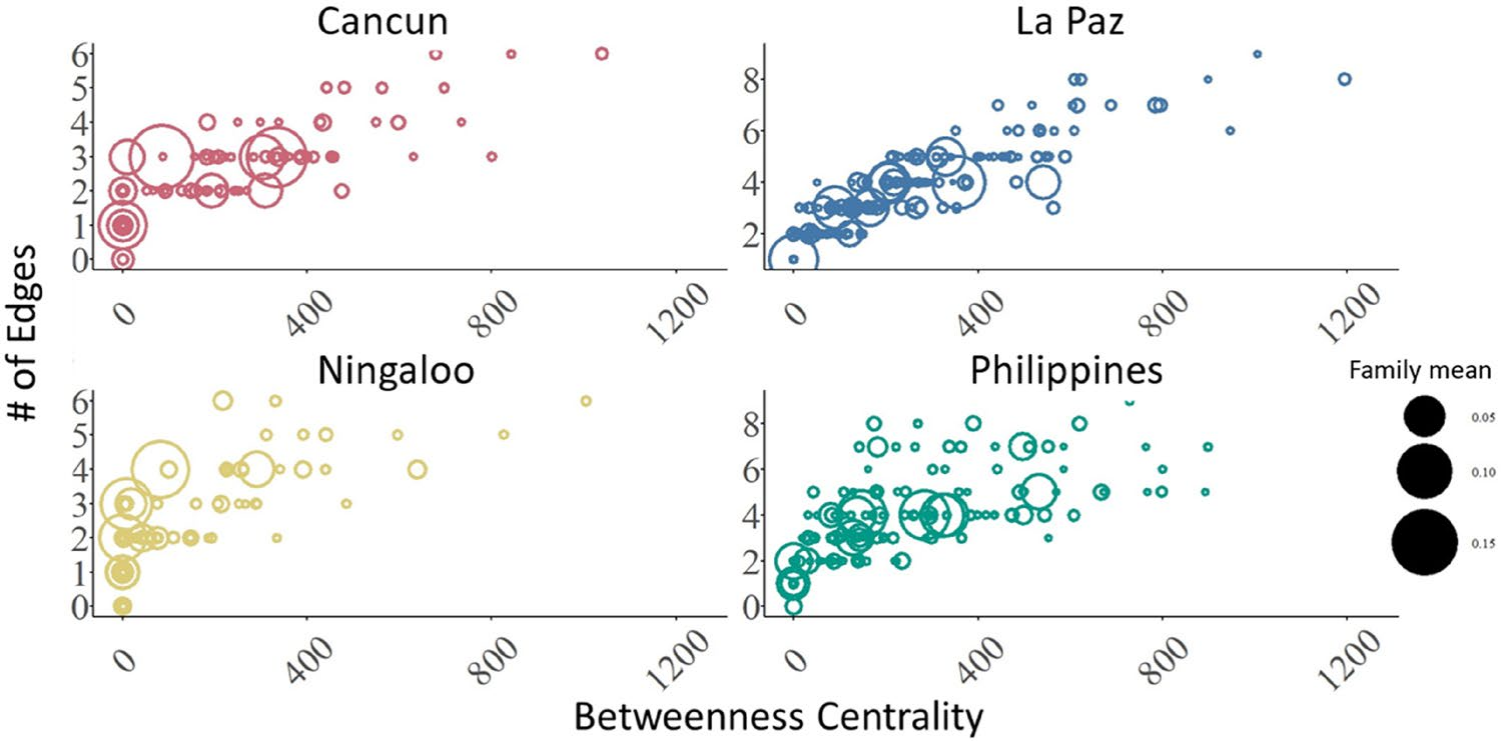
The distribution of betweenness centrality scores for each microbial family plotted against its number of edges for each location. The size of the symbols corresponds to the mean relative abundance of each microbial family.

To determine emergent microbiome patterns, network architecture was compared across four of the five aggregations (Tanzania was excluded due to low sample size) (Fig. 3a, b). The number of taxonomic families (nodes) in each network ranged from 101 (Cancun and Ningaloo) and 160 (Philippines) with mean node degrees of 2.1, 2.4, 3.8, and 3.9 (mean number of co-occurrences with other microbial families; Ningaloo, Cancun, Philippines and La Paz, respectively; Fig. 3a). Degree distribution curves of the microbiomes at the four locations were consistent (Fig. 3b). As there is no agreed upon way for comparing networks distances^17^ and our objective was to compare the statistical properties of each network to one another, we made null model comparisons. Each microbiome-created network was compared to 5000 bootstrapped *G*(*m*, *n*) random networks generated based on the number of nodes and edges for each location (Two-sample Kolmogorov Smirnov: *p* > 0.05 in 19,997 out of 20,000 total bootstraps; Fig. 3b). These microbiome networks from all aggregations shared similar global network characteristic (i.e., the number of co-varying microbial families), each consistent with that of an Erdos–Renyi network. In other words, each microbiome network was consistent with the null model comparison. Interestingly, sub-network structure of each microbiome-created network displayed modularity, an attribute that random networks do not possess. Modularity scores of the microbiome-created networks (Cancun: 0.70; La Paz: 0.58; Ningaloo: 0.71; Philippines: 0.60) were higher than the upper 95% confidence threshold for modularity scores of the randomised networks (upper CI; Cancun: 0.69; La Paz: 0.52; Ningaloo: 0.69; Philippines: 0.52), indicating a significant difference, therefore demonstrating underlying community structure in the microbiome network topology. Modularity indicates some microbial families have stronger co-occurrence with a set of microbial families relative to the rest of the microbes, therefore forming clusters within the network. Each network had ~ 11 clusters of microbial families (Cancun—12; La Paz—11; Ningaloo—12; Philippines—10). However, family membership within a cluster was not consistent across locations. We also determined whether the importance of microbial families, measured as betweenness centrality (those with greater number of edges) corresponded with the relative abundance of the microbial family (Fig. 4). Interestingly, increases in the number of edges did not correspond with an increase in relative abundance of reads in each microbial family; a consistent pattern at each location. Therefore, microbes that are of low relative abundance in the microbiome play a disproportionately large role in microbiome architecture, suggesting these are keystone microbiome species.

### Metagenome assembled genomes (MAGs) indicate a core group of whale shark epidermal microbes that occur across the world

**Figure 5:**
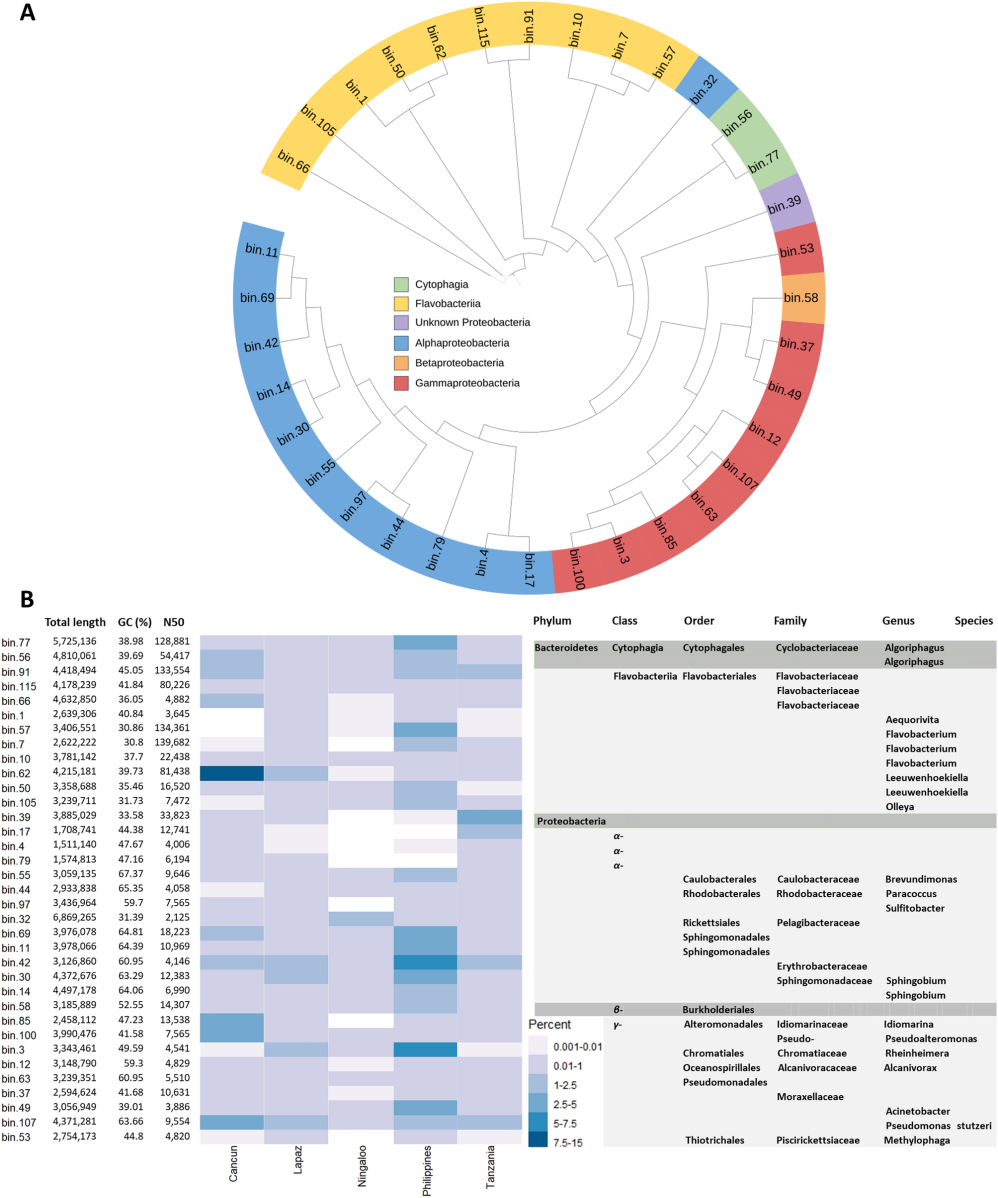
The 35 most complete metagenome assembled genomes (MAGs). (**A**) MAG phylogenetic placement relative to other MAGs with leaf colour indicating Class level taxonomic assignment. (**B**) Table and heatmap include MAG statistics, with the colors corresponding to the mean relative percent of reads from metagenomes at each location contributing to the MAGs and the hierarchical taxonomic identify of each MAG. Shading of the taxonomic identify of each MAG is based on microbial classes.  Heatmap was generated using pheatmap v. 1.0.12 (https://cran.r-project.org/web/packages/pheatmap/index.html) in the R package.

To further assess the variation in important taxa across host microbiomes, we constructed Metagenome Assembled Genomes (MAGs). A total of 118 MAGs were generated, 35 of which were high quality with completeness of  ≥ 70% and contamination ≤ 5% (Fig. 5a, b)^18^. There were 268 contigs > 50,000 bp and the longest contig was 430,630 bp. The contribution of reads to the MAGs was high across all aggregations (Philippines—64.2%; Cancun—54.4%; Tanzania—38.7%; La Paz—28.5%; Ningaloo—11.1%). MAGs were annotated to five microbial classes including Alpha-, Gamma-, and Beta-proteobacteria; Cytophagia; and Flavobacteria (Fig. 5a). MAG-bin 39 was only annotated to the Proteobacteria phyla while only a single MAG (Bin 107—*Pseudomonas stutzeri*) could be annotated to the species level, suggesting microbial members in the whale shark microbiome are quite novel. There were several MAGs in which all aggregations contributed reads equally (e.g., Bin 91- Flavobacteriaceae, Bin 107—Pseudomonadaceae, Bin 42—Erythrobacteraceae) and others where a single location over contributed (e.g., Bin 62; Cancun—Flavobacteriaceae, Bin 100; Cancun—Pseudoalteromonadaceae, Bin 3; Philippines—Chromatiaceae). Of the 35 MAGs, 25 were found across all aggregations, while eight were missing from Ningaloo and two each from Philippines and Cancun. All 35 MAGs were represented in La Paz and Tanzania whale shark microbiomes. The family identity of the MAGs corresponds with several of the most relative abundant families identified through short read annotations, including, Flavobacteriaceae, Pseudoalteromonadaceae, and Pseudomonadaceae. Alteromonadaceae, the most abundant short read annotated family, was not represented in the high-quality MAGs suggesting this family may have high diversity at the species level, with species in relatively low abundance.

### Functional metabolic characterization of the MAGs

**Figure 6:**
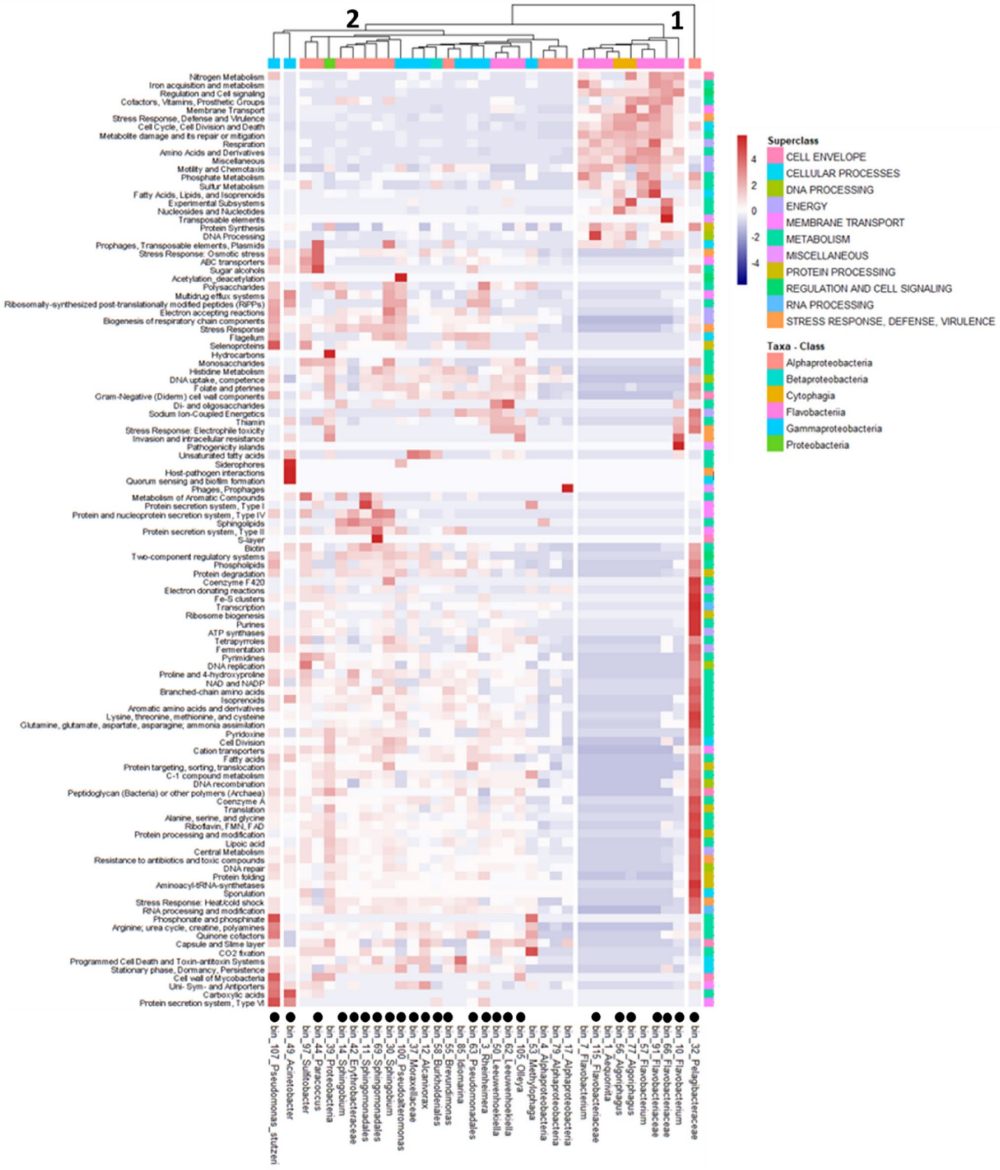
Heatmap of the 35 high quality MAGs and the relative abundance of genes associated with specific metabolic functions. Red indicates a higher abundance of genes whereas blue a lower representation within each functional Subsystem obtained from the annotation of the MAGs in the PATRIC database. Colors along the top x-axis corresponds to the taxonomic class each MAG was assigned and colors along the y-axis correspond to the Superclass each Subystem belongs. Black dots along the bottom x-axis represent those MAGs found in all locations. Heatmap was generated using pheatmap v. 1.0.12 (https://cran.r-project.org/web/packages/pheatmap/index.html) in the R package.

Clustering the gene abundance within functional subsystems defined five primary groups of MAGs (Fig. 6). Ecogroup 1 was a Bacteroidetes cluster with MAGs identified to Flavobacteria and Cytophagia classes and mostly present on whale sharks at all locations (6 of 9 MAGs). Bin 7 was however not found at Ningaloo, and bins 1 and 57 were not found at Cancun. The overrepresented gene functions in the Bacteroidetes group included nitrogen, iron acquisition, amino acid and motility and chemotaxis functions, suggesting this group’s role in trace nutrient metabolisms. Ecogroup 2 was more diverse with MAGs being identified as Alpha-, Beta-, Gammaproteobacteria, and Flavobacteriia. Again, the majority of MAGs in this cluster were distributed across the four locations (16 of 23 MAGs). Gene functions overrepresented in this group included metabolism of aromatic compounds; type I, II, and III secretion systems; and sphingolipids. Carbon metabolism, including mono-, di- and oligo-saccharides utilization were also overrepresented, suggesting this groups role in carbon metabolisms. There were three MAGs that did not cluster, indiating each had unique gene functions. MAGs of these groups were annotated as Pelagibacteraceae (Bin 32), *Acinetobater* genera (Bin 49) and *Pseudomonas sturtzeri* (Bin 107); each bin was present at all locations. Pelagibacterceae had an overrepresentation of housekeeping genes such as a range of amino acid metabolisms, carbon transport, and NAD to NADP pathways and interestingly, lacked photorhodopsin genes. *Acinetobater* had an overrepresentation of genes associated with host–pathogen interactions, quorum sensing, siderophores, multidrug resistance efflux pumps, carbonic acids, and protein secretion system type VI. *Pseudomonas sturtzeri* had an overrepresentation of genes involved in polysaccharide metabolism, stress response, multidrug resistance efflux pumps, toxin systems, phosphonate, selenoproteins, transporter genes, and flagella for motility.

## Discussion

The microbiome is increasingly viewed as more than the sum of its parts, and here we corroborate this view that microbiomes from discrete groups of whale sharks from around the world have consistent microbiome architectural characteristics. We confirm that the pattern of emergent architecture arises independently of microbiome diversity and composition. This suggests a fundamental relationship between the host and its microbiome that modulate microbial architecture rather than diversity alone. Disruption to these architectural properties thus, may provide an early indicator of dysbiosis or unhealthy microbiomes. Further, two microbial ecogroups were identified across all locations and defined as the abundant core despite some MAGs only being present at some locations. Therefore, potential gene functions from the two ecogroups may help stabilise or facilitate consistent network architecture by mineralizing highly abundant substrates in the whale shark microbiomes.

Similar network architectures within the microbiome across whale shark aggregations indicates self-organisation within these communities, and therefore similar ecological niches^19, 20^. Self-organization is not dependent upon the taxonomic identities of important community members (those with high number of predicted interactions within the network), consistent with results found in bioreactor experiments that maintained carbon cycling activities^12^. Therefore, the whale shark microbiomes are likely performing similar functions at each location, suggesting an association with the host. Further, network architecture is inherently hierarchical^11^, therefore characterising attributes of network levels, including at the whole network, sub-network, and node level reveal ecological change in microbiome functioning. For instance, the network complexity of soil microbial communities decreased with increased cropping intensity^21^ and rhizosphere networks became more organized and complex as the plants matured^22^. Therefore, the consistency of whale shark microbiome networks, regardless of location, suggest that similar ecological processes are shaping the epidermal microbiomes of the world's largest shark; perhaps a phenomenon driven by a host feature such as the filtering effects of the mucus secreted from the epidermal surface and/or of the dermal denticles^23^.

Complex microbiomes form cross-feeding networks which are expected to mediate sub-network structure, for instance modularity^24^. Whale sharks of the different aggregations consistently had modularity, or subnetwork structure of the epidermal microbiome despite the distinct geographical locations. Modularity suggests niche differentiation that results from strong selection, such as host filtering^25^, or microbe-microbe interactions in the form of cooperation and/or competition^12^. In addition, networks showed that important microbial families, defined as those with many interactions, are in low abundance. In bioreactor experiments, when the microbial communities stabilised, the community became highly connected and keystone (highly connected) microbes were in low abundance^8^. While the role of the keystones was not explicitly examined, we do suggest this group has a disproportionally large impact on total community structure by performing specialized metabolic processes^26^. Experimental evidence from a multiplicative community culture demonstrated that keystone microbes had specialized metabolisms, including cellulose and chitin degradation, each metabolicially expensive to utilize as an energy source^27^. Therefore, microbes occupying the keystone position are crucial to network structure and further research will reveal the role of these microbes in the whale shark epidermal microbiome.

The abundant core was identified by assemblying the single reads into Metagenome Assembled Genomes (MAGs). The number of MAGs characterized in our study (35 high quality) was similar to two other studies focused on shark microbiomes, one which found 54 high/medium quality MAGs from the skin surface of leopard sharks (*Triakis semifasciata*)^28^ and another that found 27 MAGs from feacal samples of two shark species^29^. These abundant core microbes may be supporting microbiome architectural patterns by providing metabolic processes. For instance, abundant microbial groups cultured from natural rainwater-filled tree holes drove bulk processes including respiration, metabolic potential, and cell yield of the total community^27^. The abundant core of the whale shark microbiome was further partitioned into two ecogroups based on the presence of potential gene functional subsystems. MAGs within the two ecogroups exhibit variation in their abundance across the sample locations, supporting results observed in the networks that important microbial groups need not be the same taxonomic groups and that functional redundancy is occurring. Ecogroup 1 was comprised of nine MAGs identified as Flavobacteriia and Cytophagia microbes. Interestingly, microbes of these taxonomic groups are commonly associated with fish mucus microbiomes^30^ and may indicate a similar role in the epidermal microbiome. Flavobacteracaea MAGs were found from the epidermal microbiome of the leopard shark (*T. semifasciata)*^28^, suggesting this microbial family my be a symbiont of marine fishes (bony and cartilaginous). Gene pathways in Ecogroup 1 support this groups role as skin microbiome symbionts that utilize mucus. Mucus, which is secreted onto the skin surface in fishes^31^ is composed of brush-like fibers formed from glycoprotein backbones which are covered in O-linked amino acids, which are rich in essential nutrients necessary for microbial activity, including sulfur, nitrogen and phophorous^32^. Ecogroup 1 MAGs were discriminated from Ecogroup 2 by the presence of gene pathways to mineralize the abundant amino acids found in this environment. Experimentally amending seawater with fish mucus was shown to trigger rapid microbial mineralization, as evidenced by a rapid increase in ammonium^33^, demonstrating the microbial response to the components of mucus. Ecogroup 1 also had an overrepresentation of genes linked with motility and chemotaxis, suggesting this group is important in the physical establishment of the microbial community, as seen in plant-root systems^34^ and coral mucus^35^. Chemotaxis may also be used by microbes to overcome inhibitory effects of the physical patterns produced by elasmobranch dermal denticles. In modelled systems, the surface topography of shark epidermis reduced biofilm formation of medically relevant microbial groups^36^. In addion, two MAGs identified as Algoriphagus (bin 77 and 56) produce lipids that inhibit development of choanoflagellates^37^, signalling a potential role in inhibiting or competing with micro-eukaryotics and other microbial groups for space. We predict the Flavobacteriia and Cytophagia are crucial microbiome members as they were found across all locations.

The Ecogroup 2 microbes were more diverse having 25 MAGs identified as Alpha-, Beta-, and Gammaproteobacteria; and Flavobacteriia. Gene subsystem present in this ecogroup were also highly diverse, and indicative of microbes driving microbial-host and/or microbe-microbe interactions along with making available shorter sugar oligomers for cross-feeding. For instance, overrepresentation of gene classes associated with metabolism of aromatic compounds, type I, II, and III secretion systems^38^ and sphingolipids^39^ suggest microbes are interacting with the surrounding environment and utilizing host substates. Interestingly, spingolipids have been suggested to interact with receptor on the skin of rainbow trout (*Oncorhynchus mykiss*) to modulate mucosal homeostatis^40^, possibly suggesting the Alphaproteobacteria from which the sphingolipids genes were identified may have a similar role in whale shark skin, helping to modulate skin mucus. Spingolipids were also identified in the epidermal microbiomes of leopard sharks (*Triakis semifasciata*) across 3 years^41^, thus spongolipids may be an important member of the epidermal microbiome of elasmobranchs. In addition, the presence of gene subclasses representing carbon utilization and exopolymer compounds, including poly- di- and oligo-saccharide metabolisms, suggests mucolytic capacity for energy and release of other limiting nutrients within mucus. MAGs constructed from the epidermal surface of two stingray species (*Myliobatis californica* and *Urobatis halleri*) contained pathways for the breakdown of long chain carbons (i.e. polysaccharides)^42^, suggesting this is a common pathway among elasmobranch epidermal microbiome members.

The three other MAGs that did not cluster with Ecogroup 1 or 2, each posessed highly diverse gene subsystems. The most notable is the Pelagibacteraceae MAG (Bin 32), which was found at each location and is a common pelagic microbial groups^43^, therefore possibly being contamination from the surrounding water column. However, Pelagibacteraceae is in the epidermal microbiome of blacktip reef sharks (*Carcharhinus melanopterus*)^44^ and coral mucus microbiome^45^, and therefore may be an inhabitant of the whale shark microbiomes.

Diversity and compositional structure provides ecological insight of whale sharks as the host epidermal microbiome was best predicted by the whale shark aggregation, possibility revealing an environmental signature. The epidermal surface of whale sharks remains in contact with the water column, which itself hosts a distinct microbial community^23^ and serves as an environmental reservoir, or regional pool of potential microbial colonizers. The whale shark microbial communities were different than the water column microbial communities, similar to other shark microbiomes^23^. However, given their movement behaviour, whale sharks regularly expose their skin surface to environmental extremes. For instance, in the Gulf of Mexico, whale sharks move as far as 52.3 km/day^46^ and dive to great depths, exposing the microbes to temperatures ranging from 30 °C at the surface to 4 °C at depth^47, 48^. Microbiome diversity patterns in whale sharks may also be shaped by inherent aggregation effects including diet, as is seen in other fish species^49^. Whale sharks in the Philippines^50^ and Tanzania^51^ primarily eat sergestid shrimp, while La Paz whale sharks feed on copepod blooms^52^, and the aggregation near Cancun ingests fish eggs^53^. Genetic structure was also a moderate predictor of microbiome structure. Whale sharks have two inferred populations based on genetics^54^: an Atlantic and Indo-Pacific group. Drivers of the microbiome at the population level may be due to inherent host-metabolic difference, or factors that co-vary with whale shark populations, such as global regions utilized by the sharks, as discussed above. Studies in other marine species show microbiome compositions that correspond to host population structure such as in some sponges^55^, and to variation in haplotype structure within populations, such as the phytoplankton *Thalassiosira rotula*^56^*.*Whale sharks have diverse haplotype structures^57^ which may be an important predictor of microbiome structure observed across aggregations however we did not test for this relationship here.

In summary, we have identified the fundamental architecture of the epidermal microbiome on whale sharks from across the world’s oceans. Our results provide evidence for an inherent assembly process, that microbiome diversity alone has not uncovered. By characterizing fundamental microbiome patterns from the whale shark, a member of the oldest extant group of vertebrates, we reveal an emergent microbiome structure which may be ubiquitous across vertebrate organisms, including human host. Thus, microbiome architecture provides a benchmark from which to interrogate healthy microbiome structure, more likely revealing underlying microbiome-host relationships. Diversity structure of the microbiome on the other hand is more informative for identifying ecological aspects of adaptations in response to plastic attributes of the host, such as environment or diet. In addition, two ecogroups emerged that suggest two ecological niches occur in the abundant core microbiome, and these groups may provide energy to other microbial groups such as keystones that support the microbiome architecture. The MAGs were identified as microbial groups commonly found in the skin microbiome of teleost fishes, however only one could be identified to the species level, which suggests novel microbial species are occupying ecologically important abundant core niche within the whale shark microbiome.

## Methods

### Sample locations and collection methods

Microbiomes were surveyed from the epidermis of 74 individual whale sharks from several locations, globally (Fig. 1). Five aggregations were sampled across 2017–2018, that included 14 whale sharks from La Paz (24°18′31.5′′ N; 110°37′42.5′′ W) in February 2017; 19 from Cancun, Mexico (21°23′49.59′′ N; 86°37′24.16′′ W) in July 2017; six from Mafia Island, Tanzania (7°52′56.67′′ S 39°39′22.35′′ E) in November 2017; 16 from Ningaloo Reef, Australia (22° 04′32.78′′ S; 113°39′06.26′′ E) in June 2018; and 19 from Oslob, Philippines (9°30′32.3 N; 123°25′01.8) in July 2018. The low number of samples from Tanzania was due to the sharks aggregating later than predicted. Microbiomes were taken from the epidermal surface along the dorso-lateral surface in line with the first dorsal fin (Fig. 1). Samples were collected using a two-way syringe device that circulates filtered seawater (0.02 µm filter) over the skin surface before being drawn into the backside of the syringe^58, 59^. This process enables the sampling of microbes from submerged whale sharks, whilst minimizing seawater microbiome contamination. From each shark we took four syringes, resulting in approximately 180 ml of sample water that was then passed through a 0.22 µm Sterivex filter (Millipore, USA), trapping all microbial life on the filter. Sterivex filters were sealed with parafilm and stored on ice until long term storage at − 20 °C. Epidermal microbiomes were the focus of the research, because they are minimally invasive, and one specific area of the shark was examined enabling the sharks to continue feeding/swimming with minimal interruption. Animal handling and ethics were reviewed at San Diego State University through IACUC under permit APF #14-05-011D, APF #17-11-010D, APF # 18-05-007D and all methods were conducted in accordance under IACUC permits.

### DNA extraction, metagenomic construction and bioinformatic processing

Genomic material was extracted directly from Sterivex filters using a modified spin column purification protocol from Nucleospin Tissue kit (Macherey–Nagel, Allentown, PA, USA). Modification to extraction procedure included first incubating sealed Sterivex filters with 720 µl of T1 buffer and 90 µl of Proteinase K (2.5 mg/mL) at 55 °C with rotation overnight. Subsequent extraction followed manufacturer protocol. DNA was prepared for sequencing using the Accel-NGS 2s Plus DNA kit (Swift Biosciences, Ann Arbor, MI, USA) for paired-end sequencing with the Illumina MiSeq v3 600 cycles (San Diego, CA, USA). Samples were barcoded and the whale shark microbiomes were mixed in with a range of microbiome samples (e.g., kelp, fish, rays, and seagrass microbiome samples) and run on several sequencing runs by the undergraduates in San Diego State University ecological metagenomics class.^60^

The 74 raw fastq files were first quality controlled using PRINSEQ^61^ with parameters set to retain reads with a minimum length of 100 basepairs, have no ambiguous bases (N), a minimum quality score of 20 and no exact duplicates. After quality control, an average of 86.4 ± 0.8% of reads were retained. Only forward reads were annotated to generate taxonomic and gene function annotations. The taxonomic identity was assigned using Focus^62^, a tool designed for rapid annotation by matching k-mer profiles calculated from metagenomic reads to precalculated k-mer profiles of reference databased genomes (Date of reference genome database: 2018).

### Network construction

Networks were constructed to examine the microbial family level architecture of the epidermal microbiome of whale sharks from different aggregations. Networks attempt to reconstruct co-occurrence patterns based on the abundance relationships of organisms in a community. The architecture of the networks is built on the distribution of the number of connected members (degree distribution) and the formation of these members into groups (modularity)^63^. Metagenomic samples are compositional and suffer from low sample size relative to the number of co-occurrences (i.e., Species 1 to Species 2 covariation), thus to overcome these limitations, we constructed networks using the SpiecEasi algorithm (version 1.0.7)^64^ which combines transformations specific to compositional values and an underlying graphical model that assumes sparse data. Due to the large number of rare microbial groups inherent to metagenomic community datasets, we removed taxa that had less than a mean of 100 total reads across all metagenomes. Network analysis is sensitive to sample size; therefore, we excluded the Tanzania samples (n = 6). The parameter values for the SpiecEasi pipeline were ‘mb’, 150 and 5e-2 for the neighborhood inference scheme, nlambda and lamda.min.ratio, respectively. These values were selected to maximize network stability (0.05 per SpiecEasi recommendation) and obtained using the *getStability* function in the SpiecEasi package. Resulting adjacency matrices were converted to igraph objects with the *adj2igraph* function in the SpiecEasi package. Network visualisations were conducted within Gephi 0.9.2 and analysis was executed using the Python networkx package (2.6), where community detection was undertaken with Clauset–Newman–Moore modularity maximisation^65^ prior to calculating the modularity score. To ascertain whether the network statistics (such as modularity) were driven by the degree distributions or smaller scale structures in the graph, for each empirical network (four locations) we bootstrapped 5000 degree-preserved randomisations and calculated the same statistics to find their means and confidence intervals within the random ensembles.

We determined whether the network degree distributions fit classical generative models of Erdos–Renyi (also referred to as a random network) or scale-free networks. A network is considered Erdos–Renyi if an edge is equally likely to connect any two nodes, or scale free if most nodes are not highly connected but there are ‘hubs’ where a single node connects to many others, creating a power law degree distribution^66^. Therefore, to compare the empirical degree distributions to a null generative model, for each aggregation, we sampled 5000 $G(m,n)$ random graphs, with $m$ nodes and $n$ edges as per the empirical network; the 5000 networks are drawn uniformly at random from the set of all possible $G(m,n)$ graphs. Two-sample Kolmogorov Smirnov tests between the empirical and random ensemble network’s degree distribution were used to generate a *p*-value distribution to accept or reject the null hypothesis that the empirical network's degree distribution is consistent with a $G(m,n)$ random graph. Note that the properties of $G(m,n)$ random graphs are, in this case, rather similar to $G(n,p)$ random graphs as we have $m\approx \left(\genfrac{}{}{0pt}{}{n}{2}\right)p$, where $p\simeq 0.025$. A Jupyter notebook implementing the network analysis is available at: https://github.com/jcmckerral/whalesharknetworks

### Construction of metagenome assembled genomes (MAGs)

To explore whether whale sharks have a core microbiome, despite the large geographic separation among aggregations, metagenome assembled genomes (MAGs) were constructed from the forward and reverse reads of the 74 metagenomes^67^. In brief, R1 and R2 reads were concatenated for assembly into contigs and the quality of assembly checked. Contigs were then binned and the quality of each MAG checked for completeness and contamination using CheckM^68^^,^^69^. Reads from each sample were then mapped back to each MAG to identify how each sample contributed to MAG construction. Taxonomy of the MAGs and MAG gene functions were identified with PATRIC version 3.6.9 using the RAST tool kit (RASTtk)^70^.

### Statistical analysis

Metagenomes were compared using proportional abundance, a more robust approach to rarefaction^71^. Alpha diversity was compared using several metrics to evaluate how abundance and richness influenced diversity patterns. Richness (S) was calculated as the total number of families found in each sample, and evenness as the quotient of Shannon’s index (H`) and S, each calculated in Vegan version 2.5.7 with the *diversity* function. Effective diversity was calculated across samples as e^(H`)^^72^. Diversity distribution was compared across aggregations with non-parametric Kruskal test with Dunn pairwise tests using the *dunn.test* function with kw = TRUE in the package dunn.test version 1.3.5.

Dissimilarity matrices were generated with *vegdis*t function from the Vegan package, with method = ‘bray’ for Bray–Curtis dissimilarity, to test differences in taxonomic family level composition across aggregations. MDS ordination was used to visualize beta diversity patterns and permutational anova (PERMANOVA) to evaluate compositional patterns across aggregations using *adonis2* function with default settings^73^. Microbiomes were evaluated using three independent tests that included aggregation (n = 5), ocean (n = 3), and population (n = 2). Pairwise permutational anova was conducted using the package pairwiseAdonis (version 0.0.1) and the *pairwiseadonis* function. All visualizations were conducted using the GGplot package (version 3.3.3) or pheatmap package (version 1.0.12). All analyses were conducted using R version 3.6.0. Code is freely available at https://github.com/mpdoane2/whaleshark_analysis.

### Ethical approval

Animal handling and ethics were reviewed at San Diego State University through IACUC under permit APF #14-05-011D, APF #17-11-010D, APF # 18-05-007D.

### Supplementary Information

Supplementary Figures.

Supplementary Table S1.

Supplementary Table 2.

Supplementary Table 3.

Supplementary Table 4.

## Data Availability

The raw metagenomic datasets generated for this project are available under BioProject accession PRJNA808622.
