# Supplementary material for: Emergent community architecture despite distinct diversity in the global whale shark (Rhincodon typus) epidermal microbiome
Source: Sci Rep. 2023 Aug 7;13:12747. doi: 10.1038/s41598-023-39184-5 (PMC10406844; doi:10.1038/s41598-023-39184-5)
Supplement: Supplementary file 1 — Supplementary Figures. [file 41598_2023_39184_MOESM1_ESM.docx]

*
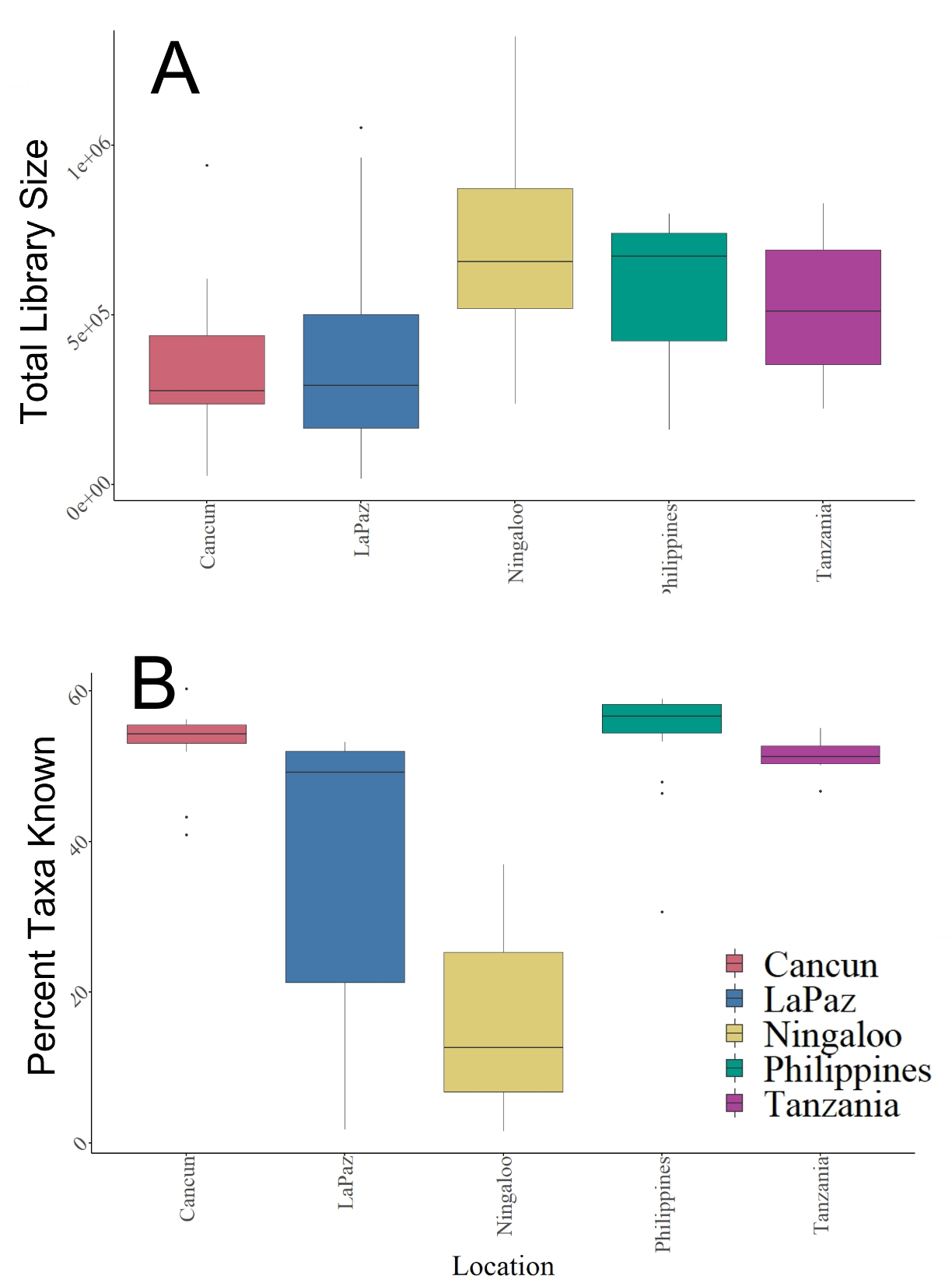
*

*Supplementary Figure S1:* (a) The median number of reads per library within each location. (b) Distribution for the percent of reads from each library with a taxonomic annotation.


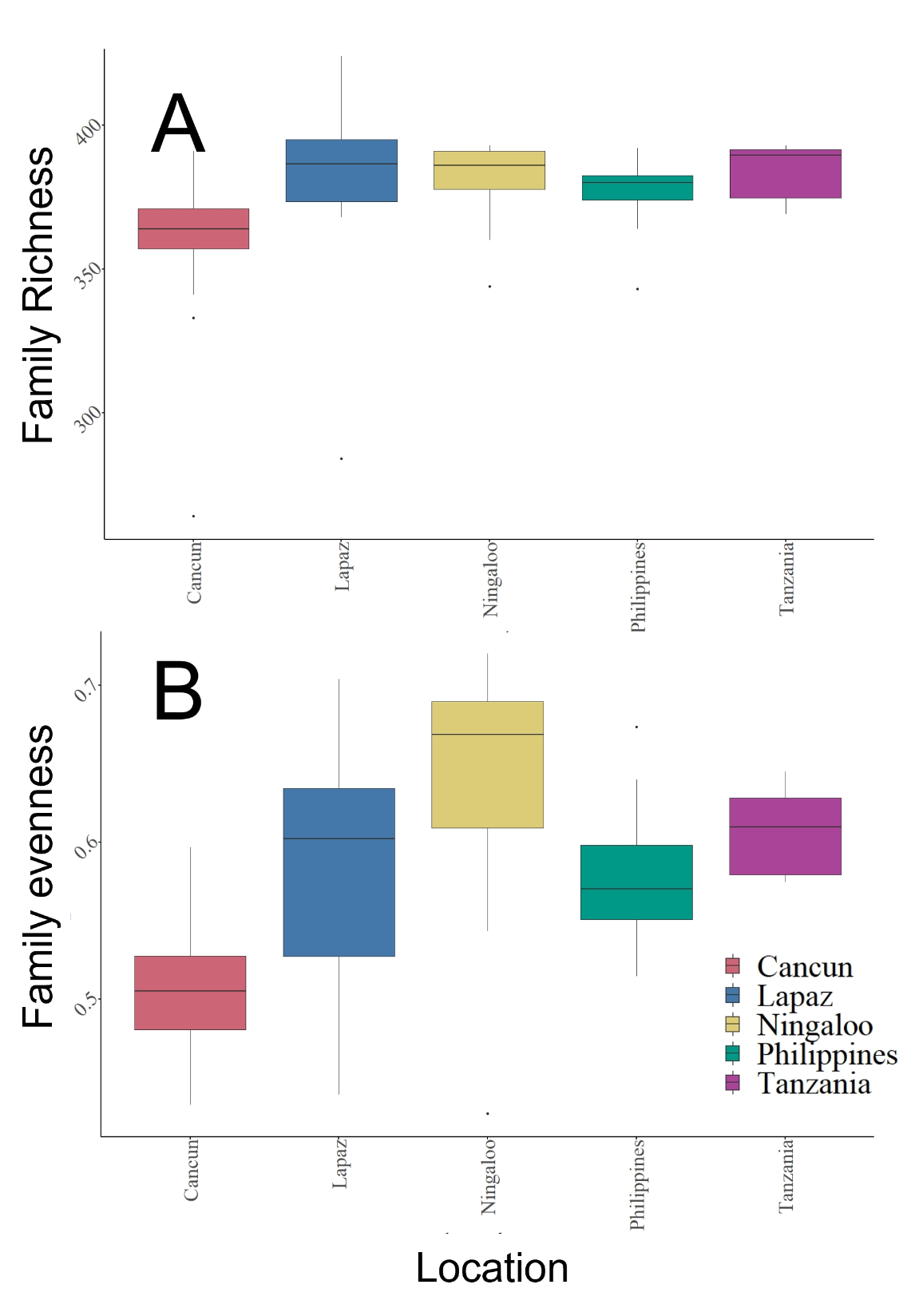


*Supplementary Figure S2:* Taxonomic microbial diversity classified to the “Family’ level calculated as (a) richness, and (b) evenness.


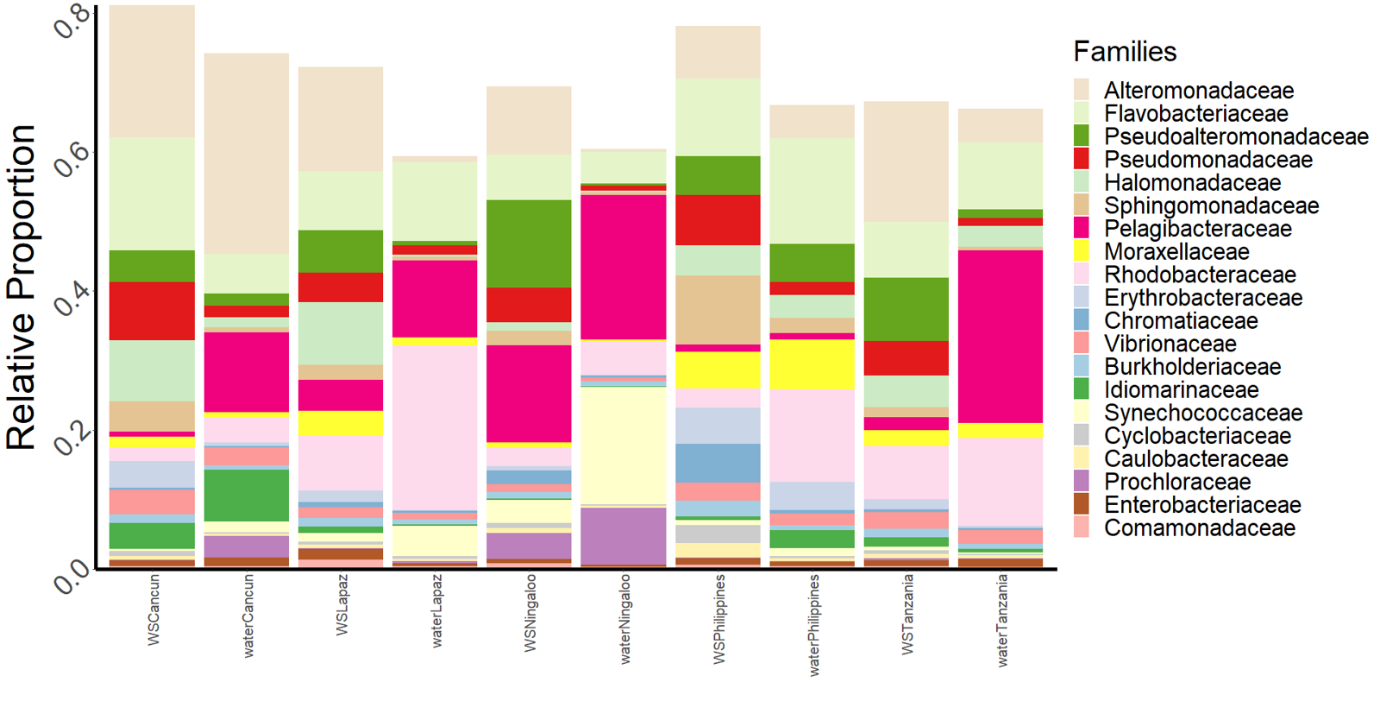


*Supplementary Figure 3*: The mean relative abundance of the top 20 microbial families from whale shark skin and the water column from each location. The water and whale shark microbiomes profiles for each location are plotted next to each other.
