# Supplementary material for: Emergent community architecture despite distinct diversity in the global whale shark (Rhincodon typus) epidermal microbiome
Source: Sci Rep. 2023 Aug 7;13:12747. doi: 10.1038/s41598-023-39184-5 (PMC10406844; doi:10.1038/s41598-023-39184-5)
Supplement: Supplementary file 2 — Supplementary Table S1. [file 41598_2023_39184_MOESM2_ESM.docx]

*Supplementary Table S1:* Sample metadata of general annotation statistics.

| **Location** | **Sample Name** | **SRA Number** | **Library size** | **Taxonomic Annotation (%)** | **Gene function Annotation (%)** |
| --- | --- | --- | --- | --- | --- |
| Cancun | Cancun 01 | SRR18160920 | 453,178 | 40.9 | 33.2 |
|  | Cancun 02 | SRR18160918 | 492,227 | 53.9 | 45.2 |
|  | Cancun 03 | SRR18160877 | 236,401 | 54.3 | 45.0 |
|  | Cancun 04 | SRR18160915 | 276,646 | 53.8 | 45.0 |
|  | Cancun 05 | SRR18160852 | 475,041 | 52.2 | 42.3 |
|  | Cancun 06 | SRR18160921 | 606,163 | 55.0 | 44.9 |
|  | Cancun 07 | SRR18160916 | 238,920 | 52.0 | 42.0 |
|  | Cancun 08 | SRR18160914 | 326,031 | 43.3 | 35.1 |
|  | Cancun 09 | SRR18160888 | 241,282 | 55.0 | 44.5 |
|  | Cancun 10 | SRR18160912 | 211,780 | 53.8 | 43.1 |
|  | Cancun 11 | SRR18160899 | 24,637 | 56.0 | 45.9 |
|  | Cancun 12 | SRR18160917 | 940,185 | 54.2 | 41.1 |
|  | Cancun 13 | SRR18160922 | 333,738 | 54.3 | 43.8 |
|  | Cancun 14 | SRR18160866 | 208,914 | 56.1 | 45.3 |
|  | Cancun 15 | SRR18160919 | 426,744 | 60.3 | 48.8 |
|  | Cancun 16 | SRR18160853 | 242,159 | 55.0 | 44.9 |
|  | Cancun 17 | SRR18160913 | 94,117 | 52.4 | 41.1 |
|  | Cancun 18 | SRR18160855 | 248,194 | 56.3 | 44.8 |
|  | Cancun 19 | SRR18160910 | 304,232 | 56.0 | 44.8 |
| La Paz | La Paz 03 | SRR18160902 | 136,573 | 50.6 | 40.4 |
|  | La Paz 04 | SRR18160903 | 1,051,392 | 49.9 | 40.3 |
|  | La Paz 06 | SRR18160905 | 291,275 | 52.8 | 44.5 |
|  | La Paz 07 | SRR18160907 | 285,155 | 52.2 | 44.1 |
|  | La Paz 08 | SRR18160908 | 235,164 | 51.8 | 44.1 |
|  | La Paz 09 | SRR18160900 | 426,966 | 43.5 | 34.7 |
|  | La Paz 10 | SRR18160906 | 963,594 | 53.2 | 13.0 |
|  | La Paz 11 | SRR18160901 | 497,571 | 49.2 | 38.6 |
|  | La Paz 12 | SRR18160909 | 18,066 | 41.0 | 30.8 |
|  | La Paz 13 | SRR18160904 | 396,647 | 52.4 | 39.1 |
|  | La Paz 14 | SRR18160911 | 139,803 | 40.3 | 31.3 |
| Ningaloo | Ningaloo 01 | SRR18160889 | 519,428 | 9.6 | 7.2 |
|  | Ningaloo 02 | SRR18160885 | 238,066 | 14.7 | 12.2 |
|  | Ningaloo 03 | SRR18160875 | 544,902 | 37.0 | 25.2 |
|  | Ningaloo 04 | SRR18160892 | 382,177 | 30.2 | 20.6 |
|  | Ningaloo 05 | SRR18160891 | 896,736 | 31.4 | 20.1 |
|  | Ningaloo 06 | SRR18160887 | 1,319,842 | 21.9 | 16.7 |
|  | Ningaloo 07 | SRR18160879 | 1,189,870 | 6.8 | 4.1 |
|  | Ningaloo 08 | SRR18160886 | 638,004 | 23.7 | 17.4 |
|  | Ningaloo 09 | SRR18160881 | 510,618 | 10.6 | 6.8 |
|  | Ningaloo 10 | SRR18160884 | 630,503 | 3.0 | 1.8 |
|  | Ningaloo 11 | SRR18160876 | 707,456 | 20.2 | 12.1 |
|  | Ningaloo 12 | SRR18160880 | 866,034 | 6.7 | 4.5 |
|  | Ningaloo 13 | SRR18160883 | 515,662 | 4.9 | 3.0 |
|  | Ningaloo 14 | SRR18160890 | 676,497 | 1.6 | 1.0 |
|  | Ningaloo 15 | SRR18160882 | 894,225 | 31.0 | 21.8 |
|  | Ningaloo 16 | SRR18160878 | 699,249 | 10.4 | 6.6 |
| Philippines | Philippines 01 | SRR18160857 | 784,848 | 53.3 | 42.4 |
|  | Philippines 02 | SRR18160872 | 427,448 | 46.4 | 34.9 |
|  | Philippines 03 | SRR18160873 | 726,836 | 30.7 | 22.1 |
|  | Philippines 04 | SRR18160874 | 545,575 | 47.9 | 36.4 |
|  | Philippines 05 | SRR18160871 | 672,917 | 55.1 | 42.3 |
|  | Philippines 06 | SRR18160865 | 742,862 | 58.3 | 45.8 |
|  | Philippines 07 | SRR18160864 | 735,329 | 56.9 | 44.0 |
|  | Philippines 08 | SRR18160856 | 548,429 | 58.3 | 45.7 |
|  | Philippines 09 | SRR18160862 | 350,237 | 58.6 | 44.4 |
|  | Philippines 10 | SRR18160867 | 697,284 | 57.4 | 46.1 |
|  | Philippines 11 | SRR18160854 | 418,819 | 56.7 | 44.1 |
|  | Philippines 12 | SRR18160860 | 437,654 | 55.7 | 42.9 |
|  | Philippines 13 | SRR18160858 | 762,371 | 54.5 | 42.2 |
|  | Philippines 14 | SRR18160868 | 798,116 | 58.2 | 44.9 |
|  | Philippines 15 | SRR18160869 | 776,716 | 55.7 | 43.0 |
|  | Philippines 16 | SRR18160863 | 738,416 | 57.2 | 44.1 |
|  | Philippines 17 | SRR18160859 | 343,210 | 54.4 | 41.8 |
|  | Philippines 18 | SRR18160861 | 399,505 | 59.0 | 45.7 |
|  | Philippines 19 | SRR18160870 | 161,137 | 58.8 | 46.0 |
| Tanzania | Tanzania 01 | SRR18160893 | 828,584 | 46.7 | 33.9 |
|  | Tanzania 02 | SRR18160898 | 224,105 | 53.1 | 40.6 |
|  | Tanzania 03 | SRR18160895 | 587,152 | 50.2 | 36.4 |
|  | Tanzania 04 | SRR18160896 | 434,743 | 51.1 | 37.6 |
|  | Tanzania 05 | SRR18160894 | 726,386 | 51.6 | 37.5 |
|  | Tanzania 06 | SRR18160897 | 326,027 | 55.1 | 45.1 |
