# Supplementary material for: Emergent community architecture despite distinct diversity in the global whale shark (Rhincodon typus) epidermal microbiome
Source: Sci Rep. 2023 Aug 7;13:12747. doi: 10.1038/s41598-023-39184-5 (PMC10406844; doi:10.1038/s41598-023-39184-5)
Supplement: Supplementary file 3 — Supplementary Table 2. [file 41598_2023_39184_MOESM3_ESM.docx]

Supplemental Table 2: Microbial family effective diversity comparison across sampling locations using pairwise Dunn test. * indicates a significant difference between the two locations.

| Pairwise Dunn test | | | |  | |  | |
| --- | --- | --- | --- | --- | --- | --- | --- |
|  | Cancun | Lapaz | Ningaloo | | Philippines | |  |
| Lapaz | 3.30934 |  |  | |  | |  |
|  | 0.0023* |  |  | |  | |  |
| Ningaloo | -5.33058 | -1.75728 |  | |  | |  |
|  | 0.0000* | 0.0657 |  | |  | |  |
| Philippines | -3.03989 | 0.509206 | 2.423892 | |  | |  |
|  | 0.0039* | 0.3816 | 0.0154* | |  | |  |
| Tanzania | -2.58602 | -0.09303 | 1.248568 | | -0.47992 | |  |
|  | 0.0121* | 0.4629 | 0.1513 | | 0.3507 | |  |
