# Supplementary material for: Emergent community architecture despite distinct diversity in the global whale shark (Rhincodon typus) epidermal microbiome
Source: Sci Rep. 2023 Aug 7;13:12747. doi: 10.1038/s41598-023-39184-5 (PMC10406844; doi:10.1038/s41598-023-39184-5)
Supplement: Supplementary file 4 — Supplementary Table 3. [file 41598_2023_39184_MOESM4_ESM.docx]

Supplemental Table 3: Microbial family richness (S) comparison across sampling locations using pairwise Dunn test. * indicates a significant difference between the two locations.

| Pairwise Dunn test | | |  |  |
| --- | --- | --- | --- | --- |
|  | Cancun | Lapaz | Ningaloo | Philippines |
| Lapaz | -3.5975 |  |  |  |
|  | 0.0008* |  |  |  |
| Ningaloo | -3.60403 | 0.120871 |  |  |
|  | 0.0016* | 0.4519 |  |  |
| Philippines | -2.47899 | 1.314028 | 1.233663 |  |
|  | 0.0165* | 0.1888 | 0.1811 |  |
| Tanzania | -2.88558 | -0.17251 | -0.26824 | 1.16809 |
|  | 0.0065* | 0.4795 | 0.4928 | 0.1734 |
