# Supplementary material for: Emergent community architecture despite distinct diversity in the global whale shark (Rhincodon typus) epidermal microbiome
Source: Sci Rep. 2023 Aug 7;13:12747. doi: 10.1038/s41598-023-39184-5 (PMC10406844; doi:10.1038/s41598-023-39184-5)
Supplement: Supplementary file 5 — Supplementary Table 4. [file 41598_2023_39184_MOESM5_ESM.docx]

Supplemental Table 4: Microbial family evenness comparison across sampling locations using pairwise Dunn test. * indicates a significant difference between the two locations.

| Pairwise Dunn test | | |  |  |
| --- | --- | --- | --- | --- |
|  | Cancun | Lapaz | Ningaloo | Philippines |
| Lapaz | 3.24929 |  |  |  |
|  | 0.0029* |  |  |  |
| Ningaloo | -5.18137 | -1.67673 |  |  |
|  | 0.0000* | 0.078 |  |  |
| Philippines | -2.94937 | 0.532532 | 2.361232 |  |
|  | 0.0053* | 0.3715 | 0.0182* |  |
| Tanzania | -2.53376 | -0.08622 | 1.19393 | 0.49038 |
|  | 0.0141* | 0.4656 | 0.1661 | 0.3466 |
